# Ultrawide-field fluorescein angiography features in patients with anterior uveitis

**DOI:** 10.1038/s41433-024-03012-5

**Published:** 2024-03-12

**Authors:** Nam V. Nguyen, Enny Oyeniran, Alexander Zeleny, Michelle Chen, Noha A. Sherif, Eleni Konstantinou, Susan Vitale, H. Nida Sen, Shilpa Kodati

**Affiliations:** 1 National Eye Institute, National Institutes of Health, Bethesda, MD USA; 2 College of Medicine, University of Nebraska Medical Center, Omaha, NE USA; 3 Department of Ophthalmology, George Washington School of Medicine, Washington, DC USA; 4 Howard University College of Medicine, Washington, DC USA; 5 University of North Carolina School of Medicine, Chapel Hill, NC USA

**Keywords:** Autoimmune diseases, Autoimmune diseases

## Abstract

**Purpose:**

To evaluate the utility of ultrawide-field fluorescein angiography (UWFFA) in patients with anterior uveitis by investigating the detection of retinal vascular leakage (RVL) and the subsequent implications on disease diagnosis and management.

**Study design/materials and methods:**

Patients, who were referred to the National Eye Institute (NEI) for evaluation of anterior uveitis and underwent UWFFA imaging at the initial visit, were included in this study. The electronic medical records of eligible patients were reviewed. The UWFFA images were assessed for severity of retinal vascular leakage, presence of macular leakage, and optic disc leakage by a two-grader system, and intergrader agreement was calculated using the κ-value. Patients with altered diagnoses and management attributable to UWFFA results were noted.

**Results:**

A total of 93 eyes of 63 patients were included in the study. Of 93 eyes, 31 (33.3%) eyes had RVL on UWFFA, with 26 (28.0%) eyes and 5 (5.4%) eyes showing mild and moderate-severe RVL, respectively. Twenty-five (26.9%) eyes showed macular leakage, and 7 (7.5%) eyes showed optic disc leakage. The κ-values ranged from 0.85 – 0.87 indicating excellent intergrader agreement. Of the 31 eyes with RVL, the diagnosis was changed to anterior/intermediate uveitis for 9 (29.0%) eyes and to panuveitis for 4 (12.9%) eyes. Systemic treatment was escalated in 5 patients.

**Conclusion:**

Our results suggest that UWFFA imaging is useful in detecting subclinical posterior involvement in patients with anterior uveitis. Moreover, UWFFA results in altered diagnosis and treatment approaches in a portion of patients.

## Introduction

Uveitis is the third leading cause of preventable blindness in the United States with an estimated annual 30,000 new cases of legal blindness [1, 2], accounting for up to 10-15% of cases of blindness in the country [2–4]. Uveitis refers to a group of heterogeneous inflammatory disorders and can be classified based on anatomical involvement into anterior, intermediate, posterior, or panuveitis [5], with anterior uveitis being the most prevalent form [6]. Although anterior uveitis (AU) refers to inflammation in the anterior chamber of the eye, a few previous studies to date have demonstrated retinal vascular leakage (RVL) on fundus fluorescein angiography in patients with AU [7–12].

Conventional fundus fluorescein angiography (FA) has been essential in diagnosing, monitoring, and managing posterior segment involvement and RVL in uveitis; however, a limited field of view compromises its ability to assess the peripheral retina [13]. Prior studies have demonstrated that peripheral vascular leakage (PVL) on FA is important as it may influence the assessment of disease activity and alter treatment decisions in patients with uveitis [14, 15]. The introduction of ultra-widefield imaging in the 2000s has transformed the scope of retinal imaging and the ability to assess the peripheral retina and detect PVL. Utilizing confocal scanning laser ophthalmoscopy, ultrawide-field imaging can capture up to a 200-degree field of view of the retina in a single image [8, 13].

To the best of our knowledge, few studies to date have utilized ultrawide-field fluorescein angiography (UWFFA) to routinely evaluate posterior segment involvement and RVL in patients with anterior uveitis [7–9, 11]. In this retrospective study, we aimed to evaluate the utility of ultrawide-field fluorescein angiography (UWFFA) in patients with anterior uveitis by investigating the presence of RVL and subsequent changes in diagnosis and management.

## Materials and methods

The study was conducted in compliance with the Declaration of Helsinki and was approved by the Institutional Review Board of the National Institutes of Health. Patients who were referred to the National Eye Institute (NEI) for evaluation of anterior uveitis and underwent UWFFA imaging at the time of their initial visit were included in the study. Patients with clinical evidence of intermediate uveitis (vitreous cells, snowballs, snowbanks), posterior uveitis (including chorioretinal lesions), panuveitis, scleritis, or vessel sheathing were excluded from the study. Patients with significant retinopathy or other retinal diseases such as diabetic retinopathy, retinal vein occlusion, and age-related macular degeneration were also excluded from the study. A total of 93 eyes of 63 patients met the inclusion criteria and were included in this study.

### Demographics

The electronic medical records of eligible patients were reviewed. Age, sex, and race/ethnicity at the initial visit were documented for all patients in the study. The diagnosis and etiology of anterior uveitis was determined by a review of medical records provided by the referring ophthalmologist and those recorded at the NEI, as well as laboratory evaluation conducted at the NEI (including laboratory testing and anterior chamber fluid testing, when applicable). The disease’s duration was defined as the period from the first documented symptomatic episode of anterior uveitis to the initial visit at the NEI. Treatment that patients were receiving at the time of the initial encounter was recorded, including topical steroids, topical nonsteroidal anti-inflammatory (NSAID) agents, oral steroids, and immunomodulatory therapies. Anterior chamber (AC) cells were quantified prior to dilation using the Standardization of Uveitis Nomenclature (SUN) Working Group classification [5]. Active disease was defined as at least +0.5 AC cells on examination. Best corrected visual acuity (BCVA), central subfield thickness (CST), and presence or absence of cystoid macular edema (CMO) on spectral-domain optical coherence tomography (Cirrus HD-OCT 5000, Carl Zeiss Meditec AG, Jena, Germany) at the visit were also documented.

### Ultra-widefield fluorescein angiography acquisition and assessment

All UWFFA images were obtained by using the Optos 200Tx (Optos PLC, Dunfermline, UK) ultra-widefield retinal imaging system, which was able to capture a 200-degree field of view of the retina. In addition to those standard images, late-phase images were obtained at 5–10 min for both eyes. Eyes with poor quality or uninterpretable images were excluded from the study.

**Fig. 1:**
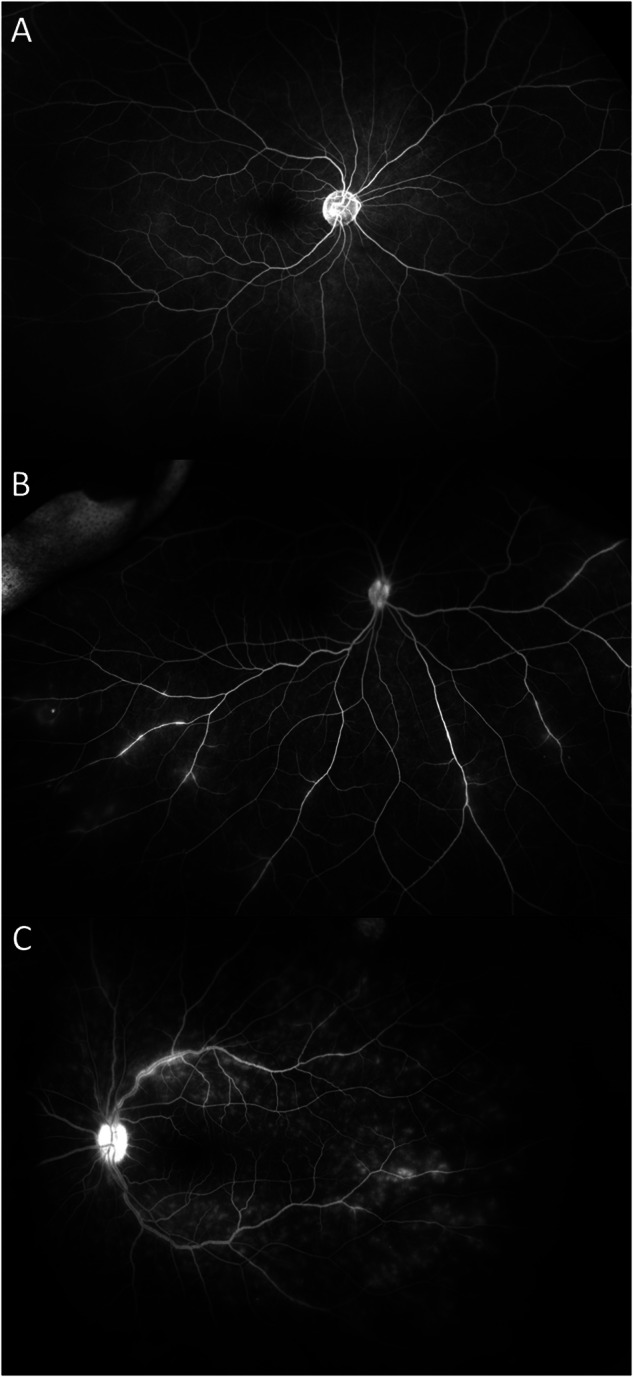
Example images of vascular leakage with different severity levels. Late phase ultrawide-field fluorescein angiography demonstrated absent (**A)**, mild (**B**), or moderate-severe (**C**) vascular leakage.

Masked grading of UWFFA images was performed independently by two ophthalmologists (EO and EK). The graders had not participated in the examination of any of the included patients and were masked to all clinical information about the patients. Optic disc leakage, macular leakage, and vascular leakage were evaluated by comparing early-phase images to the optimal late-phase images. The severity of vascular leakage was classified as absent (Fig. 1A), mild (Fig. 1B), or moderate-severe (Fig. 1C). Optic disc leakage and macular leakage were graded as present or absent. In case of disagreement between two graders, a third ophthalmologist (SK) adjudicated and determined the final assessment.

### Univariable and multivariable analysis in predicting likelihood of retinal vascular leakage

Independent variables including sex (male/female), laterality (unilateral/bilateral), duration of disease (years), AC cells (0, 0.5, and 1/2), CMO on OCT (yes/no), current oral corticosteroid use (yes/no), history of biologic (yes/no), current IMT (yes/no), and current topical corticosteroids (0.5-2x/day, 3-4x/day, and >4x/day) were examined for their association with RVL on UWFFA (none versus any). Three types of topical corticosteroids were used in this study population including Durezol (difluprednate), Pred Forte (predsinolone acetate), and Lotemax (loteprednol). The frequency of dosing was converted to prednisolone acetate equivalents for the purposes of the analysis as follows: 1 drop of difluprednate is equal to 2 drops of prednisolone acetate, and 2 drops of loteprednol is equal to 1 drop of prednisolone acetate. In the first step of the analysis, logistic regression (SAS Proc Genmod) was performed on each covariable of interest (one at a time), adjusting for age and for correlation between eyes of an individual. In the second step, multivariable logistic regression, we included age and any covariate with borderline statistical significance or odds ratio magnitude of 2-fold or more in univariable models and subsequently removed covariates not found to be statistically or clinically significant.

### Statistical analysis

Microsoft Excel (Microsoft Corp., Redmond, WA, USA), and SAS version 9.4 (SAS Institute Inc., Cary, NC, USA) were used for statistical analysis. Mean and standard deviation were calculated and expressed as mean ± standard deviation. Categorical values were expressed as frequencies and percentages. Intergrader agreement was calculated using the κ-value; strength of agreement was interpreted following guidelines from Landis and Koch [16]: <0.20: poor–slight ; 0.21–0.40: fair; 0.41–0.60: moderate ; 0.61–0.8: substantial; and 0.81–1.00: almost perfect. Two-sided *t* tests were used to determine whether differences between groups were statistically significant, using a criterion of *p* < 0.05.

## Results

### Baseline characteristics

A total of 93 eyes of 63 patients were included in the study. Forty (63.5%) patients were female, and mean age was 48.0 ± 16.7 years. Of 63 patients, 29 (46.0%) patients were Black, 22 (34.9%) patients were White, 5 (7.9%) patients were Asian, 5 (7.9%) patients were Hispanic, and 2 (3.2%) patients were of unspecified race/ethnicity. Demographic characteristics and the aetiologies of anterior uveitis are summarized in Table 1. A total of 19 eyes from 13 patients had post-surgical uveitis. Of these 13 patients, 10 developed anterior uveitis after cataract surgery. The remaining 3 patients developed anterior uveitis after the following: bilateral cataract surgery in combination with iStent placement, trabeculotomy, and combination scleral buckle/ pars plana vitrectomy for retinal detachment repair. Thirty-four (54.0%) patients had a history of bilateral involvement. Mean duration of disease was 3.9 ± 5.5 years; mean logMAR visual acuities were 0.18 ± 0.35 (approximately 20/30). At the time of initial exam, 11 eyes (11.8%) were noted to have CMO on the OCT, and mean CST was 267.4 ± 56.5 µm and 471.5 ± 203.8 µm in the non-CMO and CMO group, respectively. Seven patients were receiving treatment with systemic immunomodulatory therapy (IMT), including 3 on methotrexate, 1 on adalimumab, 1 on abatacept, 1 on azathioprine, 1 on cyclosporine, and 1 on tofacitinib. Seven patients were receiving oral corticosteroids at the time of the encounter, and of these patients, four patients were receiving concurrent treatment with at least one form of IMT. All patients were on systemic IMT and/or oral corticosteroids primarily as treatment for anterior uveitis.

**Table 1:** Baseline characteristics of the study population.

Mean age (years)	48.0 ± 16.7
Sex	
Male	23 (36.5%)
Female	40 (63.5%)
Race	
African American	29 (46.0%)
Caucasian	22 (34.9%)
Asian	5 (7.9%)
Hispanic	5 (7.9%)
Unspecified	2 (3.2%)
Laterality	
Bilateral	34 (54.0%)
Unilateral	29 (46.0%)
CST (µm)	
CME group	471.5 ± 203.8
Non-CME group	267.4 ± 56.5
Etiologies	
Idiopathic	37
Post-surgical	13
HLA-B27+	4
CMV	3
Syphilis	2
HSV	2
Sarcoidosis	1
JIA	1

*CST* central subfield thickness, *CME* cystoid macular edema, *HLA* human leukocyte antigen, *CMV* cytomegalovirus, *HSV* herpes simplex virus, *JIA* juvenile idiopathic arthritis.

Thirty-three (35.5%) eyes were noted to have at least 0.5 AC cells (active group) at the initial visit. In the active group, 29 (87.9%) eyes were receiving at least one type of treatment including topical prednisolone acetate (1%), topical difluprednate (0.05%), topical NSAID, oral prednisone, and/or immunomodulatory therapy, at the time of their initial visit. Similarly, 50 (83.3%) eyes in the inactive group were receiving treatments. Nineteen (57.6%) eyes were receiving prednisolone acetate in the active group, whereas 30 (50.0%) eyes were receiving prednisolone acetate in the inactive group. The frequency of dosing was slightly higher in the active group (6.1 ± 2.4 times/daily) compared to the inactive group (3.5 ± 2.1 times/daily) although there was no statistical difference between the two groups (*p* value = 0.06).

### Ultrawide-field fundus fluorescein angiography

The primary outcome was the presence and severity of RVL on FA at initial presentation. Of 93 eyes, 31 (33.3%) eyes had RVL on the UWFFA with 26 (28.0%) eyes and 5 (5.4%) eyes showing mild and moderate-severe RVL, respectively. UWFFA features are summarized in Table 2. Of 33 eyes with active anterior uveitis, 9 (27.3%) eyes had RVL on the UWFFA images. Of 60 eyes with inactive anterior uveitis, 22 (36.7%) eyes had RVL on the UWFFA images. The mean duration of disease was 3.5 ± 3.8 years and 4.0 ± 6.2 years in the RVL group and non-RVL group, respectively. Seventeen (54.8%) eyes were receiving prednisolone acetate in the RVL group, compared to 33 (53.2%) eyes receiving prednisolone acetate in the non-RVL group. In the RVL group, 3 eyes were receiving 8 times or less/ daily, and 14 eyes were receiving 4 times or less/daily. In the non-RVL group, 3 eyes were receiving 8 times or less/daily, and 25 eyes were receiving 4 times or less/daily. There was no significant difference between the RVL and non-RVL groups with regard to the dosing of topical prednisolone acetate. The frequency of dosing was 3.2 ± 2.0 times/daily in the non-RVL group and 3.8 ± 2.3 times/daily in the RVL group (p value = 0.15).

**Table 2:** Ultrawide-field fundus fluorescein angiography features in the study population.

Primary outcome: presence of retinal vascular leakage on FA				
	Absent	Mild	Moderate-severe	*P* value
Retinal vascular leakage (eyes)	62 (66.6%)	26 (28.0%)	5 (5.4%)	
Duration of disease (years)	4.0 ± 6.2	3.5 ± 3.8		0.32
On topical prednisolone forte (eyes)	33 (53.2%)	17 (54.8%)		
**Secondary outcome: Presence of macular leakage and optic disc leakage**				
	**Absent**	**Present**		***P*** **value**
Macular leakage (eyes)	68 (73.1%)	25 (26.9%)		
Duration of disease (years)	4.7 ± 6.2	1.9 ± 2.3		0.02*
Optic disc leakage (eyes)	86 (92.5%)	7 (7.5%)		

Secondary outcomes included the presence of macular leakage and optic disc swelling on FA at the initial presentation. Out of 93 eyes, 25 (26.9%) eyes showed macular leakage, and 7 (7.5%) eyes had optic disc leakage (Table 2). Of 25 eyes with macular leakage on FA, 11 (44.0%) eyes also had cystoid macular edema on OCT. The duration of disease in the macular leakage group on FA (1.9 ± 2.3 years) was statistically shorter than the non-macular leakage group on FA (4.7 ± 6.2 years) (p value = 0.02). In eyes with moderate-severe RVL, 3 (60.0%) eyes had concurrent macular leakage and 2 (40.0%) eyes had concurrent optic disc leakage. In eyes with mild RVL, 10 (38.5%) had concurrent macular leakage, and 3 (11.5%) eyes had concurrent optic disc leakage.

Of 31 eyes with RVL, the diagnosis was changed to anterior/intermediate uveitis in 9 (29.0%) eyes, and panuveitis in 4 (12.9%) eyes. Systemic treatment was escalated in 5 patients upon completion of their visit. Of these patients, 3 patients were started on oral prednisone, 1 on methotrexate, and adalimumab 40 mg SQ injection was increased to once a week in 1 patient. The reason for systemic escalation was primarily due to active vasculitis in 2 patients, both intraocular inflammation and active vasculitis in 2 patients, and history of recurrent flares of anterior uveitis requiring chronic topical steroids in 1 patient.

### Intergrader agreement

Complete intergrader agreement between the two graders was 98.9%, 94.6%, and 93.5% for assessment of optic disc leakage, macular leakage, and RVL, respectively. The κ-values ranged from 0.85 to 0.87, indicating excellent agreement (Table 3).

**Table 3:** Intergrader agreement between two graders in assessing retinal vascular leakage, macular leakage, and optic disc leakage.

Retinal vascular leakage	
Complete agreement	94.0%
κ-value	0.87
Weight κ-value	0.85
Macular leakage	
Complete agreement	94.9%
κ-value	0.86
Weight κ-value	0.84
Optic disc leakage	
Complete agreement	99.0%
κ-value	0.93
Weight κ-value	0.87

### Univariable and multivariable analysis of retinal vascular leakage

Univariable and multivariable analysis were performed (Table 4). In the final multivariable analysis, factors associated with RVL leakage included female sex (odds ratio, 2.94; 95% CI (0.92–9.43), p = 0.07) and presence of CMO (odds ratio, 5.09; 95% CI (1.24–20.86), p = 0.02) on OCT. However, confidence intervals were wide due to small numbers within subgroups (only 11 eyes had CMO on OCT, and only 31 eyes had RVL).

**Table 4:** Univariable and multivariable analysis in predicting retinal vascular leakage on ultrawide-field fundus fluorescein angiography.

Covariate	Estimated OR	Lower 95%CL	Upper 95% CL	*p* value
**(A) Univariable analysis in predicting retinal vascular leakage on UWFFA**				
Age (per year)	*ln(OR) 0.02	−0.02	0.06	0.34
Sex	3.12	1.02	9.49	0.04
Laterality (bilat)	0.55	0.20	1.54	0.26
Duration (per year)	*ln(OR) -0.01	−0.10	0.08	0.84
AC cells (0.5 vs 0)	0.84	0.21	3.40	0.81
AC cells (1 or 2 vs 0)	0.77	0.16	3.68	0.74
CME-OCT	3.75	0.90	15.58	0.07
Current oral corticosteroids	0.50	0.06	4.50	0.54
History of biologics	1.83	0.20	16.61	0.59
Current IMT	0.55	0.06	4.83	0.59
Current topical corticosteroids	0.80	0.21	3.15	0.76
Current topical corticosteroids: 3-4x/day	1.12	0.20	6.11	0.90
Current topical corticosteroids: >4x/day	0.62	0.16	2.39	0.49
**(B) Multivariable analysis in predicting retinal vascular leakage on UWFFA**				
Sex	2.94	0.92	9.43	0.07
CME-OCT	5.09	1.24	20.86	0.02*

## Discussion

Although anterior uveitis refers to inflammation in the anterior chamber, posterior involvement has been reported in patients with anterior uveitis as evidenced by retinal vascular leakage on FA [7–12]. In this retrospective study, we demonstrated that posterior involvement can be found in patients with anterior uveitis by using UWFFA and may alter the management and treatment of the disease in a portion of patients.

In our study, 33.3% of eyes with anterior uveitis had RVL on the UWFFA, which is consistent with other reports in the literature [7, 9, 12]. Chi et al reported a 41.5% rate of RVL in eyes with anterior uveitis on Heidelberg (Heidelberg Engineering, Inc., Heidelberg, Germany) widefield fluorescein angiography [7], while Uzlu et al reported a 42.2% rate in eyes with ankylosing spondylitis (AS)-associated anterior uveitis [9]. Similarly, Yang et al reported that 39.3% of 581 eyes with active HLA-B27 anterior uveitis had RVL on conventional FA [12]. Although these studies included anterior uveitis patients with different aetiologies, the frequency of RVL remained similar across the spectrum of anterior uveitis.

The exception to this appears to be in patients with juvenile idiopathic arthritis-associated uveitis (JIA-uveitis). In their retrospective study of 37 eyes of 20 patients with JIA-uveitis, Tripathy et al. reported approximately 70% of eyes had posterior segment inflammation on wide-field fluorescein angiography (WFA) [8]. Although definitive conclusions cannot be made from this small study, these results do suggest that JIA-uveitis patients are more likely to have posterior involvement than other types of anterior uveitis. Due to the small sample of patients with JIA-uveitis in our study, we were not able to confirm these findings. However, the majority of patients in Tripathy et al. study did not have active anterior uveitis at the WFA visit [8], and this pattern was also observed in our study and other studies [9, 12]. In our study, 27.3% of eyes with active AU had RVL on the UWFFA images while 36.7% of eyes with inactive AU had RVL. This suggests that posterior involvement may not necessarily correlate with the activity status of anterior uveitis.

Our results suggest that performing UWFFA can ultimately lead to a change in diagnosis and management in a proportion of patients. Chi et al reported that topical corticosteroids were added for 12 patients (33%) with RVL but without active disease, and 3 patients (9.1%) with significant leakage received a sub-Tenon injection of triamcinolone acetonide. Similarly, Tripathy and co-authors reported that WFA led to changes in medications in 34 eyes (91.9%) in their study. A total of 13 eyes of 7 patients in our study had the diagnosis changed to anterior uveitis/ intermediate uveitis and panuveitis based on the UWFFA results, and 5 of these patients were started on systemic therapy. This underlies the importance of UWFFA in appropriately characterizing uveitis in a subset of anterior uveitis so that treatment can be initiated to effectively control inflammation.

Although a prior study demonstrated that peripheral vascular leakage does not increase the risk of CMO or impaired visual acuity at ≥1 year of follow-up in patients with uveitis [15], our multivariable analysis indicated that the presence of CMO on OCT was associated with higher odds of RVL on UWFFA, with an estimated odd ratio of 5.09 (although confidence intervals were wide due to small numbers within subgroups). This suggests that it may be worthwhile to consider performing UWFFA, particularly in patients with more severe presentations of anterior uveitis and with CMO, in order to appropriately characterize the degree and severity of retinal vascular leakage. Additionally, previous studies have also demonstrated some functional changes on electroretinography (ERG) in patients with retinal vasculitis [17–19] further indicating the clinical significance of retinal vascular leakage. In a study by Ghoraba et al, 34 eyes of 20 patients with retinal vasculitis were included in the study, and 13 (65%) eyes demonstrated delayed 3.0 photopic 30 Hz flicker timing response with 10 (50%) eyes showing severe delay [17]. In another study by Ikeda et al, patients with retinal vascular leakage also showed depressed b-wave amplitudes response on ERG [18].

Our study had several limitations, including the retrospective nature of the study and small sample size. Furthermore, as a tertiary referral center, there may have been selection bias towards more severe presentations of AU or patients with challenging diseases courses in our cohort. Lastly, the lack of longitudinal data in our study has limited our ability to study the long-term course of patients with RVL and AU.

In conclusion, our results suggest that UWFFA imaging may be useful in detecting posterior involvement in patients with anterior uveitis. Moreover, UWFFA results altered diagnosis and treatment approach in a small portion of our patients. Given the invasive nature of FA and its potential rare side effects [20], we do not recommend FA in all patients with anterior uveitis. However, it could be considered in severe or atypical cases of anterior uveitis as well as patients with CMO.

## Summary

### What was known before

- Fundus fluorescein angiography has demonstrated retinal vascular leakage in patients with anterior uveitis.
- Retinal vascular leakage on fluorescein angiography can influence the treatment decision in patients with anterior uveitis.

### What this study adds

- Patients with macular edema as shown on OCT are more likely to have retinal vascular leakage on fluorescein angiography.
- This study further demonstrated the usefulness of ultrawide-field fluorescein angiography in characterizing the extent of uveitis and informing the treatment approach for the diseases.

## Data Availability

The data that support the findings of this study are available from the corresponding author, SK, upon reasonable request.
